# Hypoxic pretreatment of adipose-derived stem cell exosomes improved cognition by delivery of circ-Epc1 and shifting microglial M1/M2 polarization in an Alzheimer’s disease mice model

**DOI:** 10.18632/aging.203989

**Published:** 2022-04-01

**Authors:** Haining Liu, Mingming Jin, Minxiu Ji, Wei Zhang, An Liu, Tao Wang

**Affiliations:** 1 Department of Geriatric Psychiatry, Shanghai Mental Health Center, Shanghai Jiao Tong University School of Medicine, Shanghai 200030, China; 2 Alzheimer’s Disease and Related Disorders Center, Shanghai Jiao Tong University, Shanghai 200030, China; 3 Shanghai Key Laboratory of Molecular Imaging, Shanghai University of Medicine and Health Sciences, Shanghai 201318, China

**Keywords:** circ-Epc1, Alzheimer's disease, adipose derived stem cells, exosomes, microglia

## Abstract

Alzheimer’s disease (AD) is the most common dementia in the world. Increasing evidence has shown that exosomes from hypoxic pretreated adipose-derived stem cells (ADSCs) could be an effective cognitive function therapeutic in AD-associated pathophysiology. However, their role and regulatory mechanism remain largely unknown. High-throughput sequencing was used to identify differentially expressed circRNAs from ADSCs or hypoxia pretreated ADSC exosomes. Luciferase reporter assays and RT-qPCR were used to investigate the relationships between circ-Epc1, miR-770-3p, and TREM2. APP/PS1 double transgenic AD model mice were then used to study therapeutic effects regarding circ-Epc1 in ADSC exosomes. BV2 cells were used to show the regulatory relationships between circ-Epc1, miR-770-3p, and TREM2 and to show how these interactions modulated phenotypic transformations and inflammatory cytokine expressions in microglia. The results showed that exosomes from hypoxia pretreated ADSCs had a good therapeutic effect on improving cognitive functions by decreasing neuronal damage in the hippocampus. High-throughput sequencing showed that circ-Epc1 played an important role in hypoxia-pretreated ADSC exosomes regarding their ability to improve cognitive functions. Luciferase reporter assays showed that TREM2 and miR-770-3p were downstream targets of circ-Epc1. Overexpressing miR-770-3p or downregulating TREM2 reversed the effects of circ-Epc1 on M2 microglia during lipopolysaccharide treatment. *In vivo* experiments showed that circ-Epc1-containing ADSC exosomes increased the therapeutic effect of exosomes by improving cognitive function, decreasing neuronal damage, and shifting hippocampal microglia from the M1 polarization to the M2 polarization stages. Taken together, the data show that hypoxic pretreatment of ADSC exosomes improved cognition by delivery of circ-Epc1 and by shifting microglial M1/M2 polarization in an AD mouse model.

## INTRODUCTION

Alzheimer’s disease (AD) is the most common form of dementia in the world, and is becoming more prevalent due to aging of the human population; it thus constitutes a major challenge to health care systems [1, 2]. The hallmark pathological feature of AD is the deposition of β-amyloid (Aβ), which has strong neurotoxicity in brain tissue, and leads to cognitive impairment [3, 4]. AD is a microglial-mediated neuroinflammatory disease. Increasing evidence has suggested that microglial activation in the central nervous system is heterogeneous, which could be categorized into disparate classes involving the M1 and M2 phenotypes. Due to the activated phenotype, microglia could generate either neuroprotective or cytotoxic effects [5]. The different microglial phenotypes vary according to disease stage and severity. The ability to control stage-specific switching of M1/M2 phenotypes at appropriate times might therefore provide therapeutic benefits to AD patients [6, 7].

Mesenchymal stem cells (MSCs) are a family of adult stem cell that can produce a large number of multivesicular bodies that are secreted in the form of exosomes. Exosomes vary in size, with diameters of approximately 30–150 nm, which can cross the blood-brain barrier. Exosomes can carry a large number of non-coding (nc) RNAs, including circRNAs to the brain by cross the blood brain barrier (BBB), to alter the physiology of this organ [8, 9]. Exosomes derived from MSCs play regulatory roles during AD [10–12]. A former study reported that when adipose-derived stem cell (ADSC) exosomes were injected intravenously, they significantly improved learning and memory capabilities, decreased plaque deposition, and Aβ levels normalized the inflammatory cytokine levels [13]. Previous studies found that the reparative functions of exosomes from MSCs are enhanced by hypoxia treatment [14, 15]. The present study showed that hypoxia-pretreated ADSC exosomes had increased treatment efficacy for AD-induced nerve damage and cognitive impairment in the hippocampus. The aim of the current study was therefore to determine the role and regulatory mechanism regarding ADSC exosomes during AD.

## MATERIALS AND METHODS

### Animals and ethics statement

We obtained APP/PS1 double transgenic mice and B6C3-Tg (APPswe, PSEN1dE9) 85Dbo/J (original species No. 004462) mice (male with 6 weeks old) from Jax Laboratory (Bar Harbor, ME, USA). Our laboratory maintained transgenic mice on a standard 12-h light/dark cycle under constant temperature with free access to water and food. The animal ethics committee at Shanghai Mental Health Center, Shanghai Jiao Tong University School of Medicine approved this study. All protocols followed the “Guide for the Care and Use of Laboratory Animals” from the National Institutes of Health (Bethesda, MD, USA), and all efforts were made to minimize the number of animals utilized and any painful experiments.

### ADSCs isolation, culture, and identification

Washed adipose tissues were harvested from healthy subjects or normal mice with phosphate-buffered saline (PBS) and minced before digestion with 0.2% collagenase I (Sigma-Aldrich, St. Louis, MO, USA) for 1 h at 37° C with intermittent shaking. The washed tissue was digested with Dulbecco’s Modified Eagle’s Medium (DMEM; Sigma-Aldrich), which included 15% fetal bovine serum (FBS, Gibco BRL, Frederick, MD, USA), and the resulting suspension was centrifuged at 1,000 rpm for 10 min to eliminate mature adipocytes. The resulting pellet was resuspended in DMEM containing 15% FBS, 100 U/mL penicillin, and 100 μg/mL streptomycin and cultured at 37° C with 5% CO_2_. When the ADSCs reached 80%~90% confluency, they were detached using 0.02% ethylenediaminetetraacetic acid (EDTA)/0.25% trypsin (Sigma-Aldrich) for 5 min at room temperature and then replated. For phenotypic analyses, we used fluorescein isothiocyanate (FITC)-conjugated CD29, CD44, CD90, CD105, and vWF antibodies. An IgG-matched isotype was used as an internal control for every antibody. Normoxic ADSC cultures were incubated in 95% air (20% O_2_) and 5% CO_2_. For hypoxia induction, ADSCs were cultured in 94% N_2_, 1% O_2_, and 5% CO_2_.

### ADSCs multilineage differentiation

To validate ADSCs multilineage differentiation, third passage mouse ADSCs were cultured in adipogenic differentiation medium (Sigma-Aldrich), and were stained with Oil Red O after 2 w, or cultured in osteogenic differentiation medium (Sigma-Aldrich) and stained with Alizarin Red after 3 w.

### ADSC-derived exosome isolation and identification

After reaching 80%~90% confluency, ADSCs (from mice) were rinsed with PBS and cultured in FBS-free endothelial cell growth medium (EGM)-2MV supplemented with 1× serum replacement solution (PeproTech, Cranbury, NJ, USA) for another 2 d. The conditioned culture medium was removed and centrifuged at 300 *× g* for 10 min and then at 2,000 *× g* for 10 min to remove apoptotic cells and cellular debris. Briefly, we removed cell debris and large membrane vesicles by sequential centrifugation at 300 *× g* for 10 min, 2,000 *× g* for 10 min, and 10,000 *× g* for 0.5 h, followed by filtration through 0.22-μm syringe filters. The clear supernatant was then transferred to fresh tubes and centrifuged at 100,000 *× g* for 70 min. The supernatant was removed, and the pellet was washed with PBS to collect exosomes. We characterized exosomes via transmission electron microscopy and western blotting, and the size was determined by dynamic light scattering using nanoparticle tracking analysis (NTA; NanoSight, Malvern, Worcestershire, UK).

### Exosome tracing

The exosomes were labelled using the lipophilic dye, DiI (1,1′-dioctadecyl-3,3,3′,3′-tetramethylindocarbocyanine perchlorate; Thermo Fisher Scientific, Waltham, MA, USA) for biodistribution detection.

### Strand-specific high-throughput RNA-Seq library construction

Total RNA was extracted from ADSC exosomes (Exo) and hypoxia-pretreated ADSC exosomes (HExo) with or without hyperglycemia pretreatment using TRIzol Reagent (Invitrogen, Carlsbad, CA, USA). About 3 μg total RNA from every sample was subjected to a VAHTS Total RNA-seq (H/M/R) Library Prep Kit from Illumina (Vazyme Biotech Co., Ltd, Nanjing, China). In this way, ribosomal RNA was removed, but other types of RNAs were retained, including ncRNA and mRNA. The purified RNA was treated with 40 U RNase R (Epicenter, Madison, WI, USA) at 37° C for 3 h, followed by TRIzol purification, and RNA-seq libraries were constructed using the KAPA Stranded RNA-Seq Library Prep Kit (Roche, Basel, Switzerland); samples were then subjected to deep sequencing with an Illumina HiSeq 4000 from Aksomics (Shanghai, China).

### Exosome injections

The 2-month-old APP/PS1 mice were treated with PBS (AD), exosomes derived from ADSCs (Exo), hypoxia-pretreated ADSCs (HExo), or circ-Epc1-expressing ADSCs (circ-Epc1-Exo) monthly for 2 months (*n*=10 mice per group). For all groups, the injection volume was 100 μL. Exosomes from 1×10^9^ ADSCs were dissolved in 100 μL PBS. There was no drop-out during normal breeding during the injection period. To detect exosome presence in the brain, we euthanized three mice from each group at 5 h post-injection to examine brain slices by confocal microscopy after counterstaining with 4′,6-diamidino-2-phenylindole (DAPI).

### Morris water maze (MWM) test

The memory and learning functions were determined using the MWM [16]. The operators were blinded to the identities of all treatment groups. The apparatus consisted of a round steel pool (diameter: 122 cm; height: 60 cm) that was filled with water to 1 cm higher than the platform (diameter: 10 cm; depth: 30 cm) top. A blue curtain with cues surrounded the pool and was placed in an isolated room (20° C, 60% humidity). We maintained the water at 21° C and opacified it by adding titanium dioxide.

Testing was performed for 5 d. The first 4 d (P40–P43) comprised a place navigation (reference memory) test including 16 training trials (four trials per day for 4 d, with 30–40 min inter-trial intervals). At the start of every trial, the mice were placed in water facing the wall in various starting locations (south, north, west, or east), and were allowed 1 min to discover and 15 s to stay on top of the hidden platform. If the mouse could not locate the platform within 1 min, it was guided to and allowed to stay on the platform for 15 s. We used a video tracking system to track the swimming activity of every mouse. Escape latency, involving timing from placement into the water to staying on the platform, was tracked. We performed the spatial probe test when the platform was moved out of the pool. We placed the animal in an opposite quadrant and allowed it to swim freely for 2 min, then tracked platform crossing numbers. Data were analyzed using motion-detection software for the MWM test (Shanghai Mobile Datum Information Technology, Shanghai, China).

### BV2 cell culture and transfection

BV2 cells (Wuhan Biofavor Biotechnology Service, Wuhan, China) were maintained in DMEM (Invitrogen) supplemented with 10% FBS (Invitrogen) in an atmosphere containing 5% CO_2_/95% air. For phenotypic analyses, we transfected BV2 cells with a small interfering (si)RNA against TREM2, a circ-Epc1 overexpression plasmid, or a miR-770-3p overexpression plasmid (mimic) (GeneCopoeia, Shanghai, China) using Lipofectamine 2000 (Invitrogen) following standard procedures. Cells were used for further experimentation after 2 d, when they were exposed to lipopolysaccharide (LPS; 1 μg/mL) for 1 d before phenotypic analysis.

### RNA and miRNA extraction with real-time (RT)-PCR

Total RNA was isolated from the serum, cells, or brain tissues using TRIzol reagent. First strand cDNA was synthesized using the PrimeScript RT Master Mix (Perfect Real Time) Kit (Takara, Shiga, Japan), which was used for RT-PCR, along with reverse and forward primers and the Power SYBR Green PCR Master Mix (Life Technologies, Carlsbad, CA, USA). U6 and GAPDH were used as internal controls, and the data were analyzed using the 2^−ΔΔCt^ method.

### Luciferase reporter assays

The putative miR-770-3p binding site was cloned into the 3′-UTR of the target gene, *TREM2,* and circ-Epc1 (Wt or Mut) into the psi-CHECK vector (Promega, Madison, WI, USA) downstream of the firefly luciferase 3'-UTR or circ-Epc1 as the primary luciferase signal system with Renilla luciferase as the normalization signal. These vectors were termed TREM2-Wt/circ-Epc1-Wt and TREM2-Mut/circ-Epc1-Mut, respectively. The psi-CHECK vector provided the Renilla luciferase signal as normalization to compensate for differences between transfection and harvesting efficiencies. We performed transfection of HEK293 cells using Lipofectamine 2000 (Invitrogen). Renilla and firefly luciferase activities were detected 1 d after transfection using the Dual-Luciferase Reporter Assay System (Promega) with a luminometer (Molecular Devices, San Jose, CA, USA). Relative Renilla luciferase activities were detected following the instructions of the manufacturer (Promega).

### Immunohistochemistry (IHC) and immunofluorescence (IF) analyses

Brain tissue samples were fixed in 10% formalin solution, embedded in paraffin, and sectioned into 5 μm slices. Tissue sections were stained using a TUNEL detection kit (Zeiss, Oberkochen, Germany) for the determination of apoptosis. IF staining was conducted for Iba-I, CD11b, and CD206 to validate microglial polarization. Results were analyzed using an Axiophot light microscope (Zeiss) or a fluorescence microscope (Nikon, Tokyo, Japan), and images were photographed using a digital camera.

### ELISAs

Cell culture media was collected after the previously described treatments. ELISA kits (R and D Systems, Minneapolis, MN, USA) were used to measure the interleukin (IL)-1β, IL-6, and tumor necrosis factor (TNF)-α levels following the manufacturers ‘instructions.

### Statistical analysis

Continuous variations are denoted as the mean ± standard deviation (SD). We used one-way analysis of variance for multiple comparisons using GraphPad Prism (GraphPad Software, Inc., La Jolla, CA, USA). A P-value ≤ 0.05 was assumed to be statistically significant.

### Availability of data and materials

All data in this study are available.

## RESULTS

### ADSC-exosome characterization

**Figure 1:**
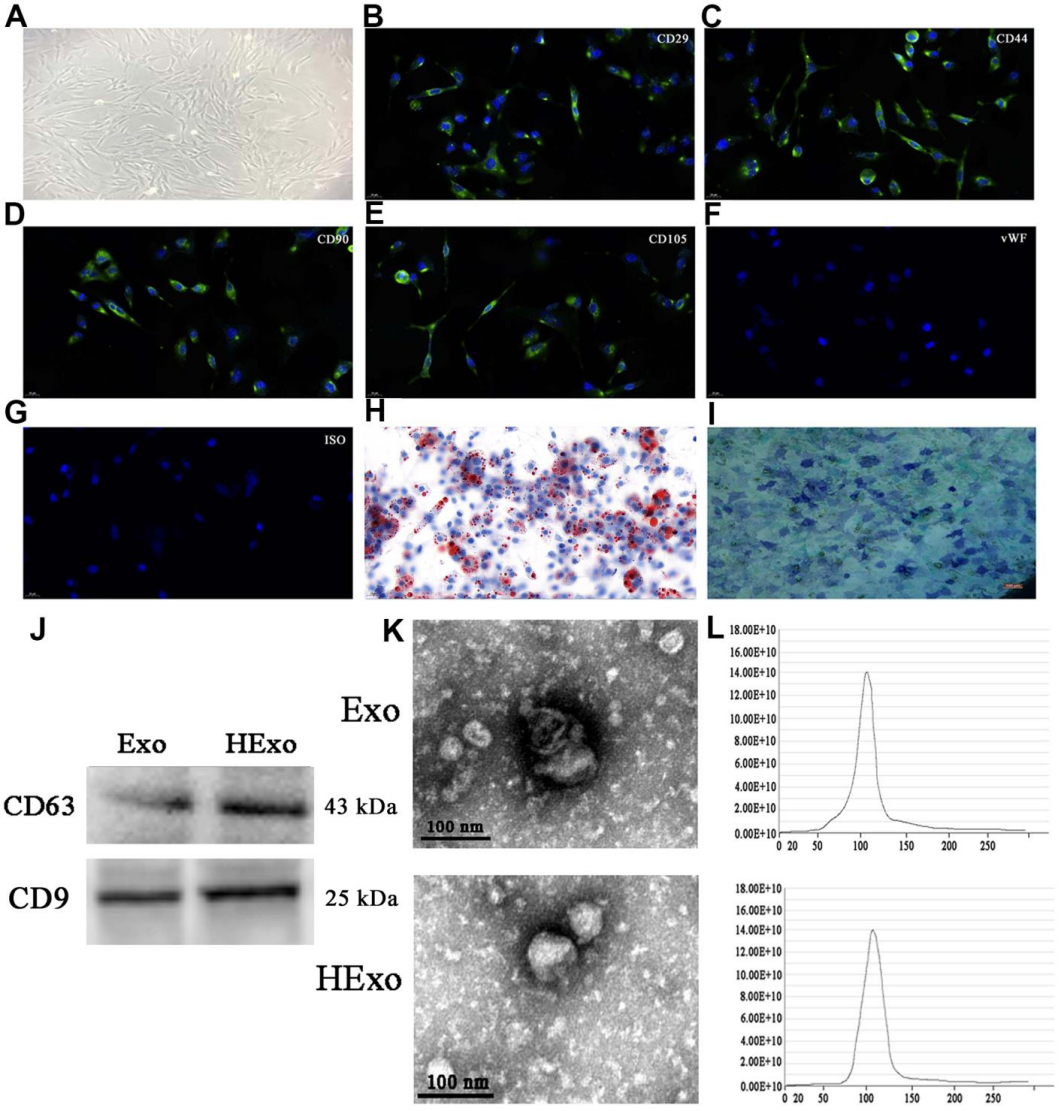
**Characterization of exosomes released by adipose-derived mesenchymal stem cells (ADSCs).** (**A**) ADSCs showed a typical cobblestone-like morphology. Scale bar: 30 μm. (**B**–**G**) Immunofluorescence staining of cell surface markers. The antibodies were labeled with fluorescein isothiocyanate (FITC, green). CD29, CD90, CD44, and CD105 were positive. The von Willebrand Factor was negative. FITC- and PE-labeled mouse IgG isotype controls are shown (magnification: 200×). Scale bar: 30 μm. (**H**, **I**) Differentiation potential of ADSCs assessed by Oil Red O (**H**) and alkaline phosphatase staining (**I**). Scale bar: 50 μm. (**J**) Western blots of CD63 and CD9 expressions in exosomes from hypoxia-pretreated or wild-type ADSCs. (**K**) Transmission electron micrographs showing ADSC-exosome morphology. Scale bar: 100 nm. (**L**) Particle size distribution and concentration of ADSC-exosomes measured by nanoparticle tracking analysis.

Previous studies reported that exosomes from ADSCs reduced Aβ pathology and neuronal cell apoptosis in a transgenic mouse model of AD [17]. Nevertheless, the underlying mechanism remains unclear. In the current study, our laboratory isolated ADSCs to confirm the typical cobblestone-like morphology (Figure 1A). IF staining was positive for mesenchymal cell markers, CD90, CD29, CD44, and CD105, and negative for endothelial marker, vWF (Figure 1B–1G). Oil Red O (Figure 1H) and Alizarin Red (Figure 1I) staining validated that ADSCs differentiated into various lineages such as osteoblasts and adipocytes.

Western blot analyses of exosome lysates showed positive expressions of exosomal proteins, CD9 and CD63, in both normal and hypoxia-pretreated ADSCs (Figure 1J). Transmission electron microscopy showed that ADSC-exosomes exhibited characteristic cup-shaped morphologies (Figure 1K). We quantified the size of ADSC-exosomes using the Zetasizer Nano (Malvern Panalytical, UK). The mean vesicle diameter was 80–130 nm (Figure 1L), which was consistent with formerly described exosomes [18]. Overall, the results strongly suggested that the nanoparticles were exosomes.

### Exosomes from hypoxia-pretreated ADSCs improved cognitive function by decreasing neuronal damage in the hippocampus

**Figure 2:**
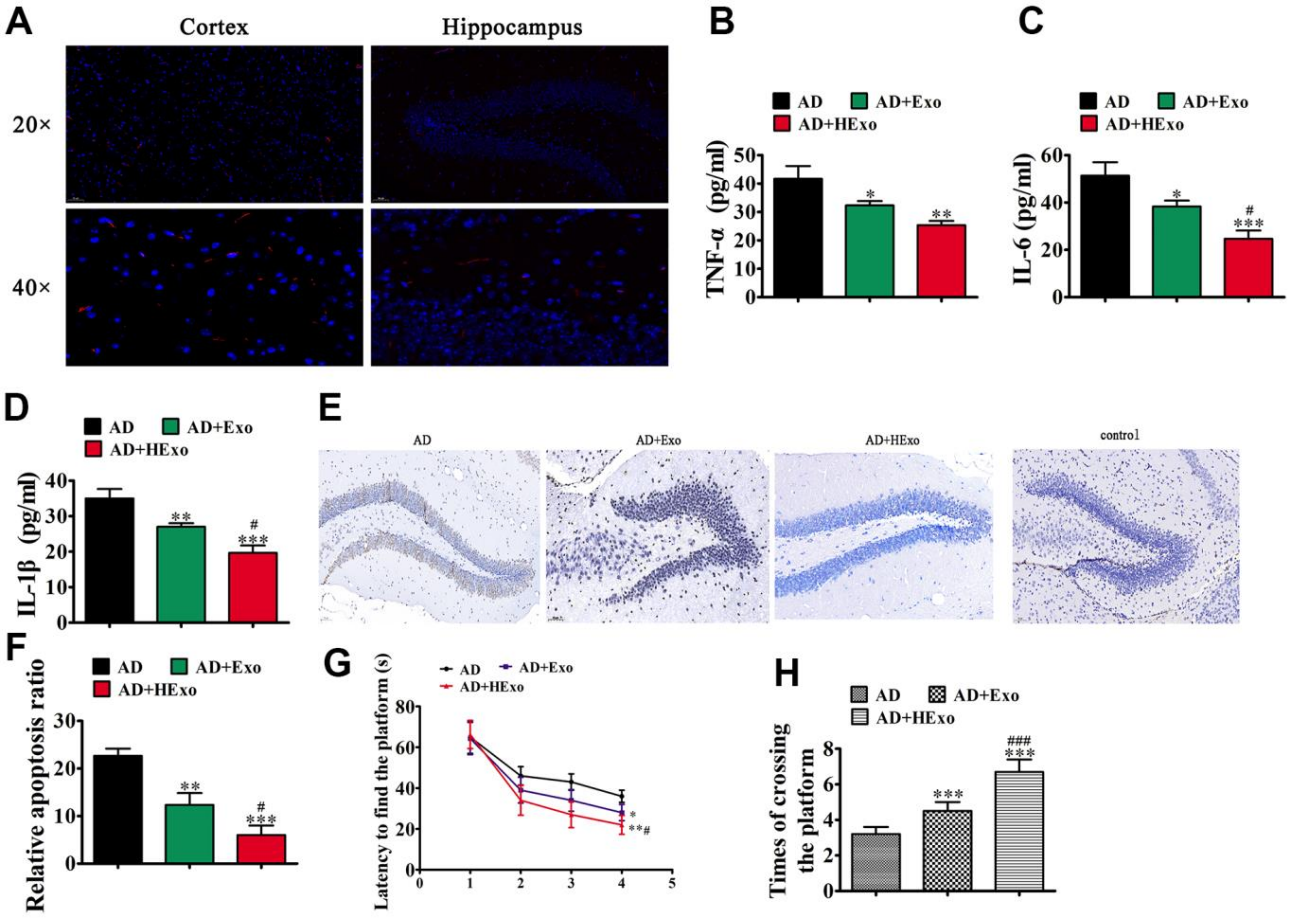
**Exosomes from hypoxia-pretreated adipose-derived mesenchymal stem cells have a more therapeutic effect at improving cognitive function by decreasing neuronal damage in the hippocampus.** (**A**) The 1,1′-dioctadecyl-3,3,3′,3′-tetramethylindocarbocyanine perchlorate-labeled exosomes are red and nuclei are counterstained with 4′,6-diamidino-2-phenylindole (blue). The injected exosomes were detected in the cortex and hippocampus. (**B**–**D**) ELISA assays showing expression of the inflammatory factors, TNF-α, IL-6, and IL-1β. Data represent mean ± SD (*n*=10). ^*^P < 0.05, ^**^P < 0.01, ^***^P < 0.001 vs. the control; ^#^P < 0.05 vs. exosomes derived from ADSCs (Exo). (**E**, **F**) Hippocampal neuron apoptosis was detected using the TUNEL assay. Data represent the mean ± SD (*n*=6). ^**^P < 0.01, ^***^P < 0.001 vs. the control; ^#^P < 0.05 vs. Exo. (**G**) Alzheimer’s disease mice exhibited a longer escape latency than exosome-treated animals. Data represent the mean ± SD (*n*=10). ^*^P < 0.05, ^**^P < 0.01 vs. the control; ^#^P < 0.05 vs. Exo. (**H**) The number of platform crossings was increased in the exosome-treated group. Data represent the mean ± SD (*n*=10). ^*^P < 0.05, ^***^P < 0.001 vs. the control; ^###^P < 0.001 vs. Exo.

To calculate the presence of exosomes derived from ADSCs in the cortex and hippocampus, brain slices were observed using a fluorescence microscope at 5 h after injection. We found DiI-labeled exosomes in the hippocampus and cortex (Figure 2A). ELISAs detected the presence of inflammatory factors, IL-6, TNF-α, and IL-1β, in brain tissues. These results showed that exosome treatment inhibited the expressions of inflammatory factors. Exosomes from hypoxia-pretreated ADSCs (HExo) had a greater therapeutic effect and decreased expressions of IL-6, TNF-α, and IL-1β (Figure 2B–2D). IHC showed that HExo suppressed nerve apoptosis in the hippocampus (Figure 2E, 2F) more than other treatments. To characterize the behavioral consequences of exosomes in the mouse model of AD, spatial learning and memory were assessed using the MWM (Figure 2G). Exosome treatment decreased the escape latency, when compared with that of untreated AD mice. HExo had a greater therapeutic effect in decreasing escape latency than normal exosomes. Furthermore, using the spatial probe test, the platform crossing number increased in the Exo especially in the HExo treatment group (Figure 2H).

### The increased therapeutic effect of hypoxia-pretreated ADSC exosomes in improving cognitive function involved circ-Epc1

**Figure 3:**
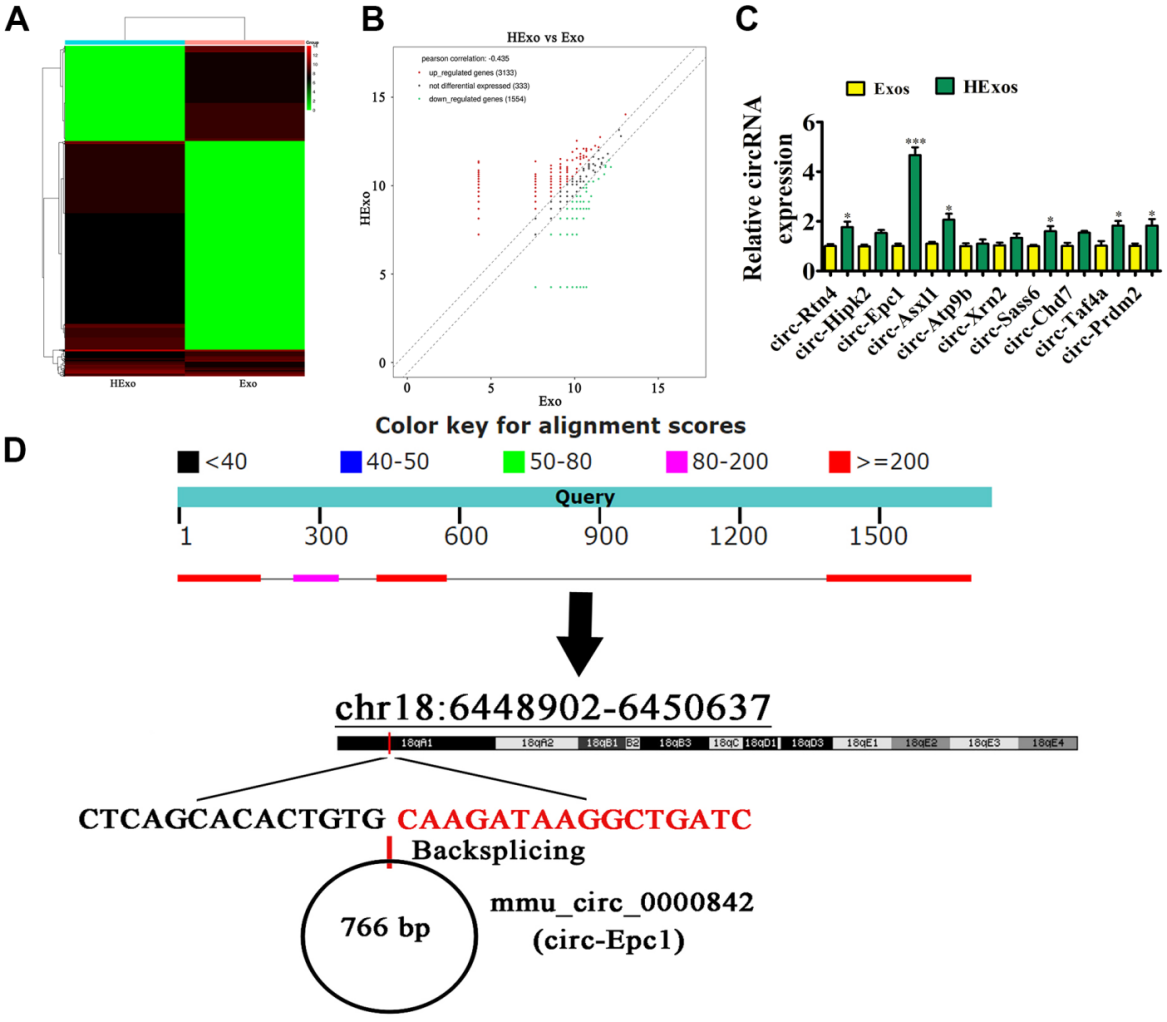
**Exosomes from hypoxia-pretreated adipose-derived mesenchymal stem cells had a greater therapeutic effect at improving cognitive function when delivering circ-Epc1.** (**A**) Heat map showing the differentially expressed circRNAs. (**B**) Volcano plot showing the upregulated and downregulated circRNAs from exosomes derived from ADSCs (Ex)o and hypoxia-pretreated ADSC exosomes (HExo). (**C**) RT-qPCR detection showing the expression of 10 upregulated circRNAs between Exo and HExo. Data are the means ± SD. ^*^P < 0.05, ^***^P < 0.001 vs. Exo. (**D**) The chromosomal location of circ-Epc1.

Former studies have reported that ncRNAs function in AD pathogenesis [19]. The present study utilized high-throughput sequencing to characterize the differentially-expressed circRNAs in Exo and HExo. The results showed that hypoxia pretreatment led to abnormal expressions of circRNAs (Figure 3A). Compared with Exo, hypoxia pretreatment led to 3,133 circRNAs being upregulated and 1,554 circRNAs being downregulated (Figure 3B). RT-qPCR showed upregulated expressions of 10 circRNAs (circ-Rtn4, circ-Hipk2, Circ-Epc1, circ-Asxl1, circ-Atp9b, circ-Xrn2, circ-Sass6, circ-Chd7, circ-Taf4a, and circ-Prdm2) between Exo and HExo. These data showed that only circ-Epc1 expression was significantly increased in HExo, when compared with Exo (Figure 3C). Bioinformatics analyses showed that circ-Epc1 was derived and cyclized from an exon of *Epc1*, which was located at chr18:6448902-6450637. Together, these data suggested that circ-Epc1 may play a role in improving cognitive functions.

### TREM2 and miR-770-3p were downstream targets of circ-Epc1

**Figure 4:**
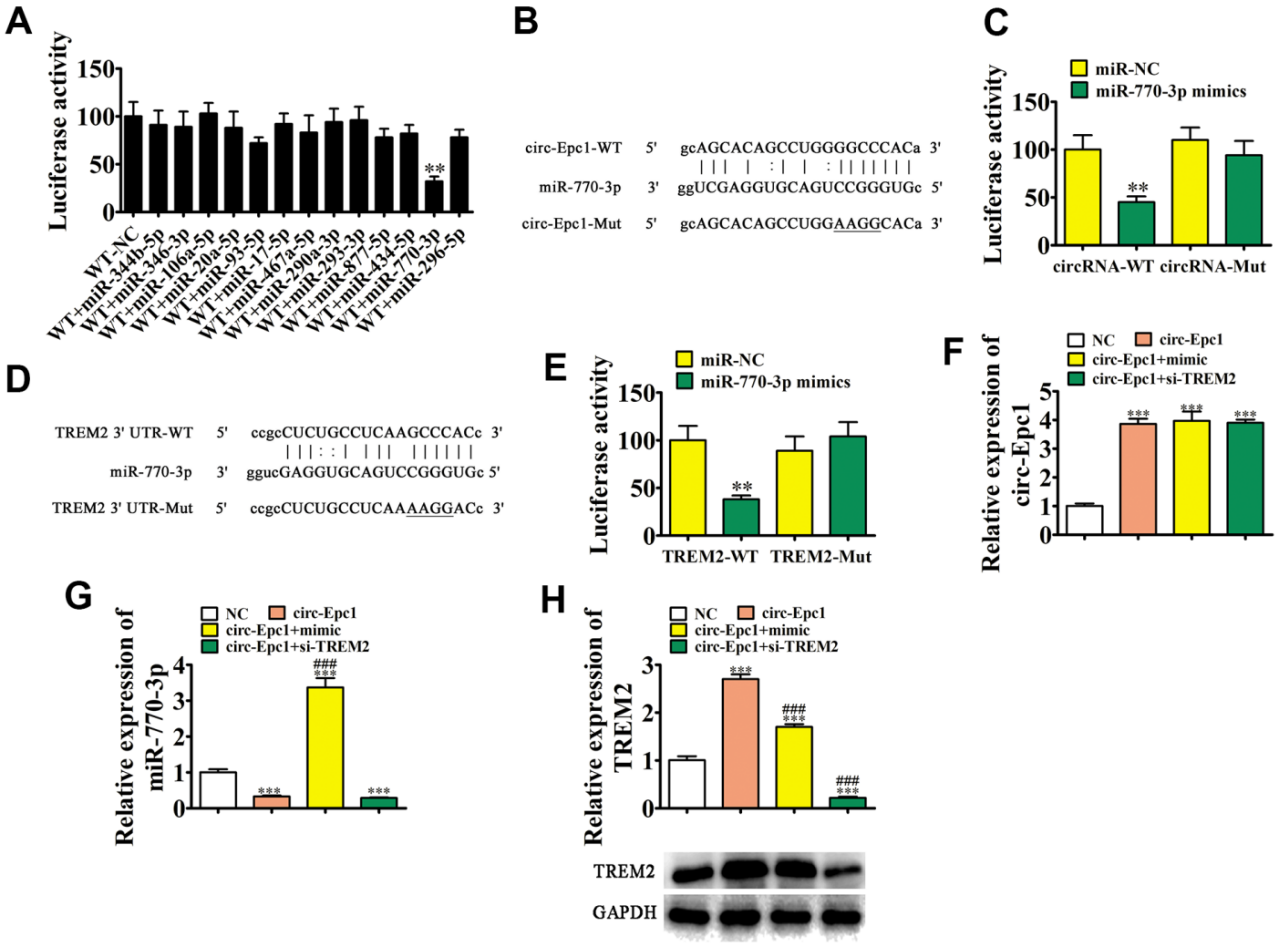
**TREM2 and miR-770-3p are downstream targets of circ-Epc1.** (**A**) Dual-luciferase reporter assays showed that co-transfecting wild-type (WT) and mimic miR-770-3p decreased the luciferase activity in HEK293T cells. Data are the means ± SD. ^**^P < 0.01 vs. WT+NC. (**B**) Predicted binding sites of miR-770-3p in circ-Epc1. The mutant version of circ-Epc1 is shown. (**C**) Relative luciferase activity determined 48 h after transfecting HEK293T cells with the miR-770-3p mimic/NC or circ-Epc1 WT/Mut. Data are presented as the means ± SD. ^**^P < 0.01. (**D**) Predicted binding sites of miR-770-3p within the 3′-UTR of TREM2. The mutant version of the TREM2 3′-UTR is shown. (**E**) Relative luciferase activity determined 48 h after transfecting HEK293T cells with the miR-770-3p mimic/NC or 3′-UTR-TREM2 WT/Mut. Data are presented as the means ± SD. ^**^P < 0.01. (**F**–**H**) RT-qPCR and western blot detection showing the expressions of circ-Epc1, miR-770-3p, and TREM2 in BV2 cells after transfection with circ-Epc1 overexpression (circ-Epc1), miR-770-3p mimic (mimic), TREM2 silencing vector (si-TREM2) individually, or in combination. Data are presented as means ± SD. ^***^P < 0.001 vs. NC; ^###^P < 0.001 vs. circ-Epc1.

A previous study found that circRNAs regulated gene expression by sponging miRNAs [20]. Bioinformatics analyses showed that circ-Epc1 interacted with miRNAs, including miR-344b-5p, miR-346-3p, miR-106a-5p, miR-20a-5p, miR-93-5p, miR-17-5p, miR-467a-5p, miR-290a-3p, mir-293-3p, miR-877-5p, miR-434-5p, miR-770-3p, and miR-296-5p. We then constructed luciferase reporter vectors containing the circ-Epc1 sequence, and transfected different miRNA mimics into HEK293 cells. The results showed that only miR-770-3p significantly decreased the fluorescein intensity, suggesting that miR-770-3p was a circ-Epc1 downstream target (Figure 4A). Luciferase reporter analysis further confirmed that miR-770-3p inhibited luciferase activity in wild-type (WT) cells but not in mutated (MUT) cell lines (Figure 4B, 4C), suggesting that miR-770-3p was the circ-Epc1 target.

Bioinformatics analysis showed that TREM2 was a miR-770-3p downstream target. To further verify the correlation regarding miR-770-3p and TREM2, WT or MUT 3′-UTR-TREM2 sequences including the miR-770-3p binding sequence were inserted into the luciferase reporter vector (Figure 4D). The transfected luciferase reporter vector was inserted into 293T cells with or without the miR-770-3p mimic. The luciferase reporter analysis showed that miR-770-3p inhibited luciferase activity in WT cells, but not in cell lines with the MUT sequence (Figure 4E), suggesting that TREM2 was a miR-770-3p target. RT-qPCR showed that circ-Epc1 expression increased in BV2 cells after transfection with a circ-Epc1 overexpression vector, while a miR-770-3p overexpression vector or siRNA against TREM2 could not reverse the circ-Epc1 expression (Figure 4F). Overexpressing circ-Epc1 inhibited miR-770-3p expression, but transfecting miR-770-3p mimics reversed and promoted miR-770-3p expression. Transfecting siRNA against TREM2 did not rescue miR-770-3p expression after silencing circ-Epc1 (Figure 4G). Our results also showed that overexpressing circ-Epc1 promoted TREM2 expression in both mRNA and protein level, while overexpressing miR-770-3p reversed this effect and promoted circ-Epc1 (Figure 4H). Together, these data showed that TREM2 and miR-770-3p were important downstream targets of circ-Epc1, and that circ-Epc1 may regulate TREM2 by sponging miR-770-3p.

### Overexpressing miR-770-3p or downregulating TREM2 reversed the effect of circ-Epc1 on shifting M2 microglial during LPS stimulation

**Figure 5:**
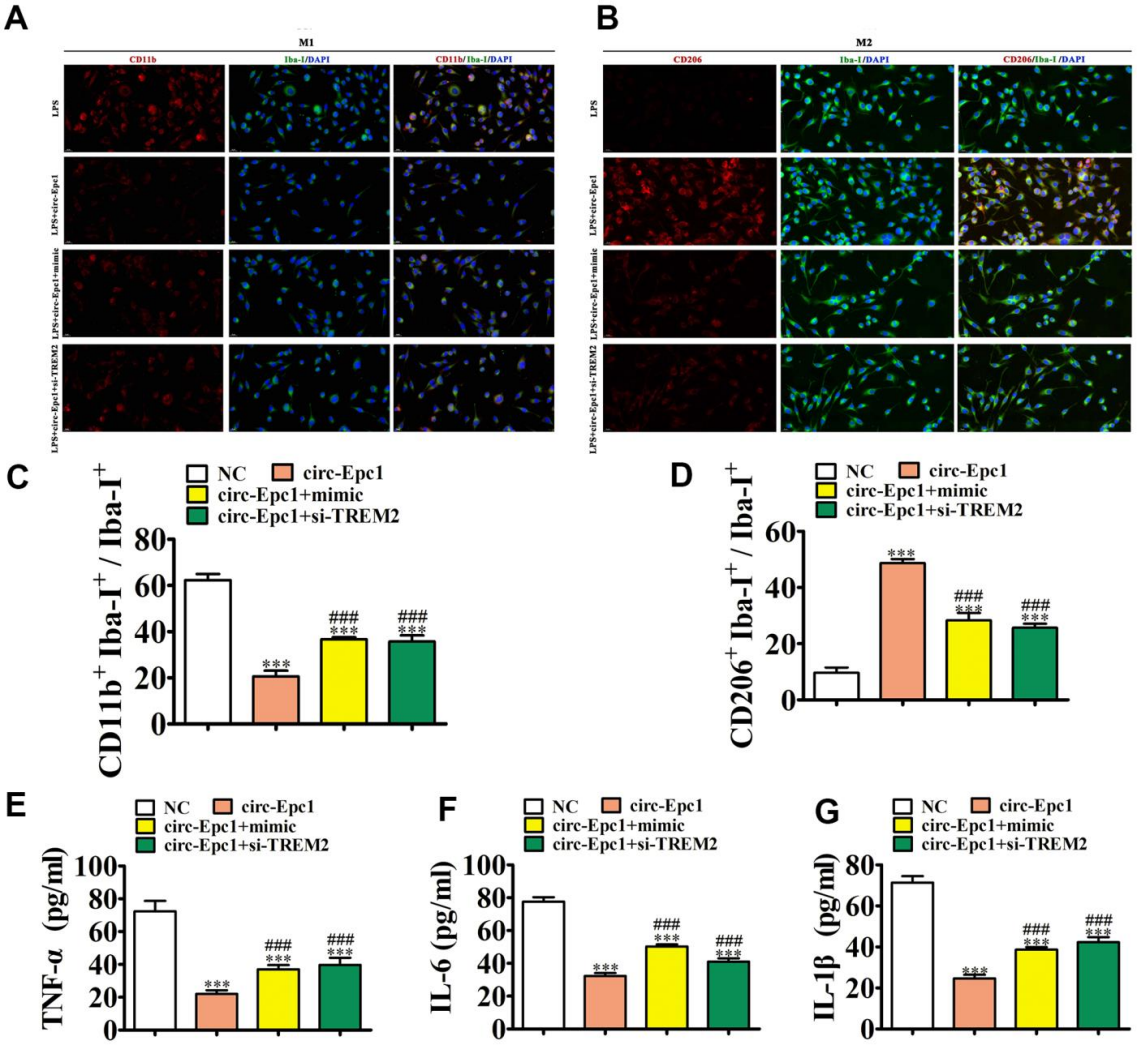
**Overexpressing miR-770-3p or downregulating TREM2 reversed the effect of circ-Epc1 on M2 microglial shifting during treatment with lipopolysaccharide (1 μg/mL).** (**A**–**D**) Immunofluorescence detection of macrophage polarization using Iba-I^+^, CD11b^+^, and CD206^+^staining. Data are presented as the mean ± SEM. ^***^P < 0.001 vs. NC; ^###^P < 0.001 vs. circ-Epc1. (**E**–**G**) ELISA detection showing expression of the inflammatory factors, TNF-α, IL-6, and IL-1β. Data are presented the mean ± SD (*n*=10). ^***^P < 0.001 vs. NC; ^#^P < 0.05 vs. circ-Epc1.

A previous study found that counteracting immunomodulation of microglia polarization reduced cognitive aging impairment and AD [21]. In this study, BV2 cells were transfected with the circ-Epc1 overexpression vector, miR-770-3p mimic, or si-TREM2, individually or in combination. After exposure to 1 μg/mL LPS for 1 d, we collected BV2 cells for phenotypic analysis. IF detection of microglial/macrophage polarization was performed using Iba-I^+^ (total microglia), CD11b^+^ (M1 microglia), and CD206^+^ (M2 microglia) as markers. The results showed that upregulating circ-Epc1 decreased M1 microglial differentiation by decreasing CD11b expression. In contrast, overexpressing miR-770-3p or downregulating TREM2 partially reversed M1 microglial differentiation following circ-Epc1 overexpression. We also found that upregulating circ-Epc1 promoted M2 microglial differentiation by increasing CD206 expression. Additionally, overexpressing miR-770-3p or silencing TREM2 partially reversed M2 microglial differentiation in circ-Epc1-overexpressing cells (Figure 5A–5D). ELISA results showed that miR-770-3p overexpression or silencing TREM2 partially reversed the inhibitory effect of circ-Epc1 on inflammatory factor expressions such as those of IL-6, TNF-α, and IL-1β (Figure 5E–5G).

### Circ-Epc1 containing ADSC exosomes (circ-Epc1-Exo) increased therapeutic efficacy and improved cognitive function by decreasing neuronal damage and shifting microglia from M1 to M2 in the hippocampus

**Figure 6:**
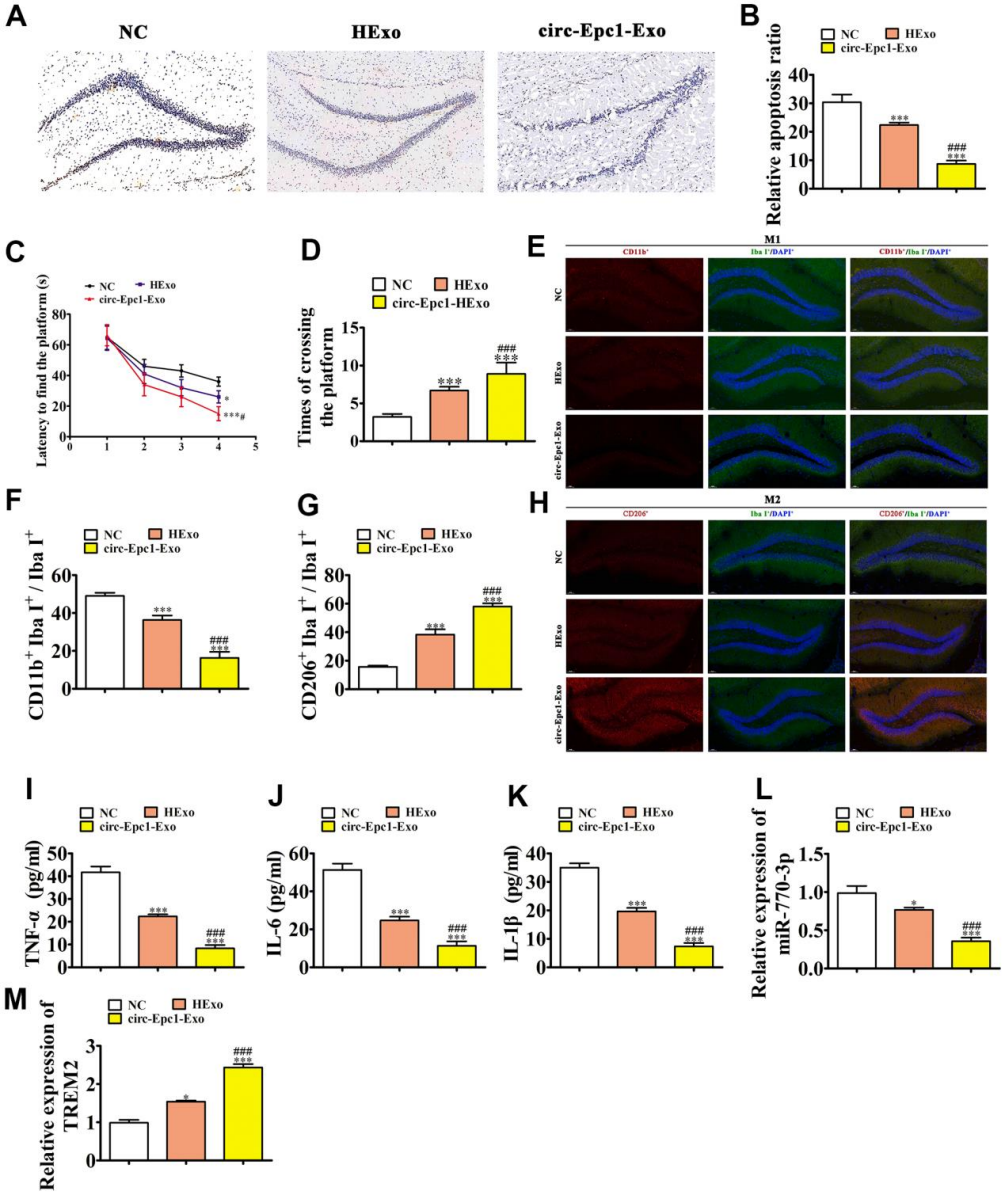
**Circ-Epc1-modified ADSC exosomes (circ-Epc1-Exo) showed increased therapeutic effects at improving cognitive function by decreasing neuronal damage and shifting hippocampal microglia from M1 to M2.** (**A**, **B**) Hippocampal neuron apoptosis was detected using the TUNEL assay. Data represent the mean ± SD (*n*=6). ^**^P < 0.01, ^***^P < 0.001 vs. the control; ^#^P < 0.05 vs. Exo. (**C**) Alzheimer’s disease mice exhibited a longer escape latency than exosome-treated animals. Data represent the mean ± SD (*n*=10). ^*^P < 0.05, ^**^P < 0.01 vs. the control; ^#^P < 0.05 vs. hypoxia-pretreated ADSC exosomes (HExo). (**D**) The number of platform crossings was increased in the exosome-treated group. Data represent the mean ± SD (*n*=10). ^*^P < 0.05, ^***^P < 0.001 vs. the control; ^###^P < 0.001 vs. HExo. (**E**–**H**) Immunofluorescence detection of macrophage polarization using F4/80^+^, CD11b^+^, and CD206^+^staining. Data are presented as the mean ± SEM. ^***^P < 0.001 vs. control; ^###^P < 0.001 vs. HExo. (**I**–**K**) ELISA results showing expressions of the inflammatory factors, TNF-α, IL-6, and IL-1β. Data are presented as the mean ± SEM. ^***^P < 0.001 vs. the control; ^###^P < 0.001 vs. HExo. (**L**, **M**) RT-qPCR showing the expressions of miR-770-3p and TREM2 in hippocampal tissues. Data are presented as the mean ± SEM. ^***^P < 0.001 vs. the control; ^###^P < 0.001 vs. HExo.

To illustrate the protective effect of circ-Epc1 on cognitive function, APP/PS1 double transgenic mice were treated with PBS, HExo, or circ-Epc1-Exo. IHC showed that circ-Epc1-Exo treatment had a greater therapeutic effect than HExo by decreasing hippocampal apoptosis (Figure 6A, 6B). To elucidate the behavioral consequences of exosome treatment in the AD mouse model, spatial learning and memory were assessed using the MWM (Figure 6C). Circ-Epc1-Exo treatment had a greater therapeutic effect than HExo by decreasing escape latency, when compared with untreated AD mice. Furthermore, using the spatial probe test, the platform crossing number increased in the HExo treatment, especially in the circ-Epc1-Exo treatment group (Figure 6D).

IF detection of microglial/macrophage polarization in the hippocampus was performed using Iba-I^+^ (total microglia), CD11b^+^ (M1 microglia), and CD206^+^ (M2l microglia) staining. The results showed that circ-Epc1-Exo treatment had a greater therapeutic effect than HExo by decreasing M1 microglial differentiation and promoting M2 microglial differentiation, through decreasing CD11b and increasing CD206 expressions (Figure 6E–6H).

ELISA assay results showed that circ-Epc1-Exo treatment had a better therapeutic effect than HExo on inflammatory factor expressions such like TNF-α, IL-1β, and IL-6 (Figure 6I–6K). RT-qPCR showed that circ-Epc1-Exo treatment also had a greater therapeutic effect than HExo by increasing miR-770-3p and decreasing TREM2 expressions in hippocampal tissues (Figure 6L, 6M).

## Discussion and Conclusions

The prevalence of AD has increased in recent years, leading to heavy burdens on families and societies. Currently, there are no efficient therapeutic drugs for AD. Stem cell exosome therapy is a possible non-pharmacological treatment because of its wide positive effect range, fewer side effects, and low economic burden [22–24]. Previous studies have found that ADSC exosome treatment reduced Aβ40 and Aβ42 levels, together with the Aβ42/40 ratio of AD cells [17]. The current study suggested that hypoxia-pretreated ADSC exosome treatment had a greater therapeutic effect than ADSC exosomes at decreasing apoptosis and inflammatory cytokines in the hippocampus. Cognitive function tests found that hypoxia-pretreated ADSC exosome treatment had greater effects than treatment with ADSC exosomes at recovering AD-induced cognitive impairments.

To verify the regulatory mechanism that explained how ADSC exosomes altered AD pathophysiology, we conducted high-throughput sequencing of exosomes from different treatment groups. The results showed that circRNAs were abnormally expressed in exosomes from hypoxia-pretreated ADSCs, when compared with untreated ADSC exosomes. RT-qPCR showed that circ-Epc1 levels were significantly increased in hypoxia-pretreated ADSC exosomes. Circ-Epc1 was derived and cyclized from part of the *Epc1* gene, which is located at chr18:6448902-6450637. Overall, our data suggested that circ-Epc1 may play a role in improving cognitive function.

CircRNAs comprise a family of noncoding single-stranded, highly stable RNA molecules that are abundant in the eukaryotic transcriptome. CircRNAs are significantly enriched in human retinal and brain tissues [19]. Previous studies have found that circRNAs contributed to AD pathogenesis [25, 26]. Another study found that circRNAs regulated gene expressions by sponging miRNAs [27]. Using a luciferase reporter system, we found in the present study that circ-Epc1 interacted with miR-770-3p. Previous studies reported that miR-770-3p expression significantly increased with aging [28]. MiR-770-3p expression can facilitate apoptosis by inducing inflammation [29]. Further studies found that miR-770-3p interacted with the triggering receptors expressed in TREM2, which was also verified by our luciferase reporter assays. TREM2 is a receptor, which is only expressed by microglia in the brain, where it changes homeostasis of the microglial immune system. Human genetic investigations have shown that loss-of-function mutations in TREM2 signaling were highly associated with an elevated risk of age-related neurodegenerative traits such as AD. TREM2 loss confers resilience to synaptic and cognitive impairment in aged mice [30].

TREM2 also ameliorates neuroinflammatory responses and cognitive impairment using the PI3K/AKT/FoxO3a pathway in AD mice [31]. Further study discovery that TREM2 can switching microglia from an M1 proinflammatory phenotype to an M2 anti-inflammatory phenotype and inhibit TLR4/NF-κB mediated inflammatory response [32]. TREM2 is one of the most highly expressed receptors in microglia (ranking 31st) and is >300-fold enriched in microglia compared to other cell types [33]. A large majority of the related studies have provided strong evidence that TREM2 dysfunction results in amyloid pathology. TREM2 has recently been demonstrated to be essential to promote the association between microglia and Aβ plaques [34].

In this study, the results showed that overexpressing circ-Epc1 promoted TREM2 and decreased miR-770-3p expressions. Overexpressing miR-770-3p also decreased TREM2 expression. TREM2 overexpression did not influence circ-Epc1 or miR-770-3p expression, suggesting that TREM2 and miR-770-3p were downstream targets of circ-Epc1. Circ-Epc1 regulated TREM2 by sponging miR-770-3p. An increasing number of studies have reported that AD induced microglial activation and transformed their polarization towards the M1 phenotype, which was associated with the production of pro-inflammatory cytokines, eventually leading to nerve cell damage in the hippocampus and cognitive impairment [35–37]. In our study, we found that circ-Epc1 expression promoted M2 microglial phenotypes during LPS stimulation. However, overexpressing miR-770-3p or downregulating TREM2 reversed the promotion effects of circ-Epc1 on shifting the M2 microglial phenotype during LPS treatment. Our *in vivo* experiments also showed that circ-Epc1-containing ADSC exosomes partially rescued AD-induced cognition impairment by shifting the microglial phenotype from M1 to M2, which resulted in decreased expressions of inflammatory cytokines and less apoptosis of hippocampal neurons.

## CONCLUSIONS

Taken together, we found that hypoxic pretreatment of adipose-derived stem cell exosomes improved cognition by delivering circ-Epc1 and shifting microglial M1/M2 polarization in the AD mice model. However, additional studies are necessary to verify and expand our findings, which might ultimately enable us to completely elucidate the mechanisms of AD.
